# A Comparative Analysis of the Efficacy of Three Plant Growth Regulators and Dose Optimization for Improving Agronomic Traits and Seed Yield of Purple-Flowered Alfalfa (*Medicago sativa* L.)

**DOI:** 10.3390/plants14152258

**Published:** 2025-07-22

**Authors:** Xianwei Peng, Qunce Sun, Shuzhen Zhang, Youping An, Fengjun Peng, Jie Xiong, Ayixiwake Molidaxing, Shuming Chen, Yuxiang Wang, Bo Zhang

**Affiliations:** 1 College of Grassland Science, Xinjiang Agricultural University, Urumqi 830052, China; psuyy521@163.com (X.P.); 18678637275@163.com (Q.S.); 18399820280@163.com (Y.A.); 18160679386@163.com (F.P.); 18108684320@163.com (J.X.); 15099441701@163.com (A.M.); 13369639628@xjau.edu.cn (S.C.); wyx9868@163.com (Y.W.); xjauzb@126.com (B.Z.); 2 Xinjiang Key Laboratory of Grassland Resources and Ecology, Urumqi 830052, China; 3 The Ministry of Education Key Laboratory of Grassland Resources and Ecology in the Western Arid and Desert Areas, Urumqi 830052, China

**Keywords:** alfalfa, agronomic traits, mepiquat chloride, principal component analysis, seed yield

## Abstract

This study evaluated the effects of different plant growth regulators and their concentration gradients on the agronomic traits, seed yield, and yield components of *Medicago sativa* L. cv. “Xinmu No. 5” alfalfa. This experiment comprised 10 treatments, including 98% mepiquat chloride (200, 250, and 300 mg/L), 5% prohexadione-calcium (150, 250, and 350 mg/L), and 5% uniconazole (50, 100, and 150 mg/L), each at three concentration levels, along with a distilled water control (CK). The results show that the 98% mepiquat chloride treatment (MCT3) significantly reduced plant height (by 22%) and internode length (by 28.3%), while increasing stem diameter, branch number, and seed yield. Plant height and internode length exhibited a significant positive correlation, and both were highly significantly negatively correlated (*p* < 0.01) with seed yield components, indicating that controlling vegetative growth can enhance seed yield. Principal component analysis (extracting four principal components with a cumulative contribution rate of 80.8%) further confirmed that the 98% mepiquat chloride treatment MCT3 (300 mg/L) was the most effective treatment for improving seed yield of alfalfa in arid regions.

## 1. Introduction

Alfalfa (*Medicago sativa* L.), globally recognized as a high-quality forage crop, is a perennial leguminous plant that is widely cultivated across the world [1]; with strong nitrogen fixation ability and well-developed root systems, Alfalfa is hailed as the “King of Pasture” and serves as a vital protein source for livestock, offering significant nutritional value and exhibiting high palatability, as well as resilience to extreme climates and salinity [2]. Following the restructuring of China’s agriculture and animal husbandry sectors, greater emphasis has been placed on improving grasslands and expanding the cultivation area of high-quality forages. This has driven a continuous growth in the demand for forage seeds, which in turn led to an imbalance between supply and demand. The supply–demand imbalance in the production capacity of alfalfa is particularly prominent [3]. Rising demand for alfalfa seeds, driven by grassland reseeding, land reclamation, and livestock industry growth, is constrained by low seed production in China, limiting domestic market supply [4]. Moreover, alfalfa is the fourth most high-value export crop in the United States [5], while China has a high dependence on imported forage [6]. Therefore, addressing the issue of low domestic seed production has become a key research priority.

Alfalfa is typically harvested 3–4 times annually, with the second and third harvests yielding the highest quantity and quality [7]. After the third harvest, yield and nutritional quality decline significantly, reaching their lowest levels in the next harvest [8], and hindering high-quality forage production. In the southern border region of China, Alfalfa’s first harvest occurs in mid-May, and the second from mid-July to mid-August, coinciding with peak production conditions: 30.9 mm precipitation, 37 °C temperatures, and 15 h sunshine. By late September to early October, reduced temperature, sunlight, and precipitation lower forage quality and yield [9]. Harvesting alfalfa seeds during this period conserves land resources, reduces economic losses, and improves seed production and quality in the region [10].

Plant growth regulators (PGRs) include synthetic chemicals and endogenous hormones such as auxins, gibberellins, and cytokinins, which regulate cellular activities through signal transduction, affecting growth, maturation, and abscission of plant organs [11]. In crops, PGRs optimize hormone levels and growth, enhancing yield, stress tolerance, and physiological traits [12]. Alfalfa yield is determined by the total dry matter produced and its distribution among various plant organs [13]. Alfalfa’s indeterminate growth can lead to overgrowth and nutrient imbalances, reducing seed yield and quality. PGR application during the vegetative phase can improve dry matter allocation, morphology, and seed yield.

Mepiquat chloride (MC), a quaternary ammonium plant growth regulator, inhibits gibberellin synthesis by blocking enzymes responsible for ent-kaurene formation [14], reducing cell elongation, and preventing excessive vegetative growth [15], consequently leading to a short plant stature and boosting crop production [16]. MC is widely used in cotton (*Gossypium hirsutum* L.) production to enhance yield by promoting the transfer of carbon reserves from stems and roots to reproductive organs [17]. In a study in cotton plants [18], MC treatment significantly affected plant height, internode length, harvest timing, and drought resistance. In another study [19], applying 60 g a.i. ha^−1^ of MC at the V4 stage reduced plant stature and lodging risk in non-oilseed sunflowers (*Helianthus annuus* L.), improving plant traits. Moreover, MC application in tomatoes (*Solanum lycopersicum* L.) at 62 g a.i. ha^−1^ improved both yield and quality [20].

Prohexadione-calcium (Pro-Ca), a calcium salt of 3,5-dioxo-4-propionylcyclohexanecarboxylate, inhibits apical dominance by combining equimolar prohexadione anions and calcium ions [21], reduces plant height, improves crop quality and yield, and lowers costs and labor [22,23]. It can also modulate physiological functions and antioxidant properties of plants, which helps them combat salinity stress [24]. Additionally, it has low mammalian toxicity and minimal crop residue formation [25], and Pro-Ca emerged as an environmentally friendly PGR [26]. In rice (*Oryza sativa* L.), exogenous Pro-Ca application enhances photosynthesis and antioxidant enzyme activity, alleviates NaCl stress, and increases tillering and grain yield [27].

S3307 (uniconazole) inhibits kaurene oxidase, which converts ent-kaurene to kaurenoic acid [28], thereby inhibiting gibberellic acid biosynthesis [29]. It belongs to the class triazole and is highly effective, with low toxicity and minimal residue-forming tendency [30]. Studies have shown that uniconazole has a significant impact on the phased growth and development of maize (*Zea mays* L.) [31]. In soybeans (*Glycine max* L.), uniconazole improves morphology, light penetration, ventilation, stunting resistance, and yield [32]. Plant growth regulators (PGRs) are increasingly recognized in agriculture for their effectiveness in regulating plant metabolism and growth, controlling apical meristem activity and excessive elongation, shaping plant architecture, and enhancing chlorophyll content [33]. The potential of PGRs to promote reproductive growth, improve plant traits, and enhance seed yield in alfalfa under arid and semi-arid conditions requires further validation.

This study systematically compared the regulatory effects of three plant growth regulators (PGRs)—98% mepiquat chloride, 5% prohexadione-calcium, and 5% uniconazole—at different concentrations on alfalfa plant architecture. By analyzing their comprehensive effects on chlorophyll content and seed yield components (e.g., number of inflorescences and pods), the optimal PGR and concentration were identified to optimize plant architecture and significantly increase seed yield. The findings provide theoretical guidance and technical parameters for the chemical regulation of alfalfa seed production in arid regions.

## 2. Results

### 2.1. Effects of Foliar Application of Different Plant Growth Regulators on Agronomic Traits and SPAD Values of Alfalfa

#### 2.1.1. Effects of Plant Growth Regulators on the Key Agronomic Traits

**Figure 1:**
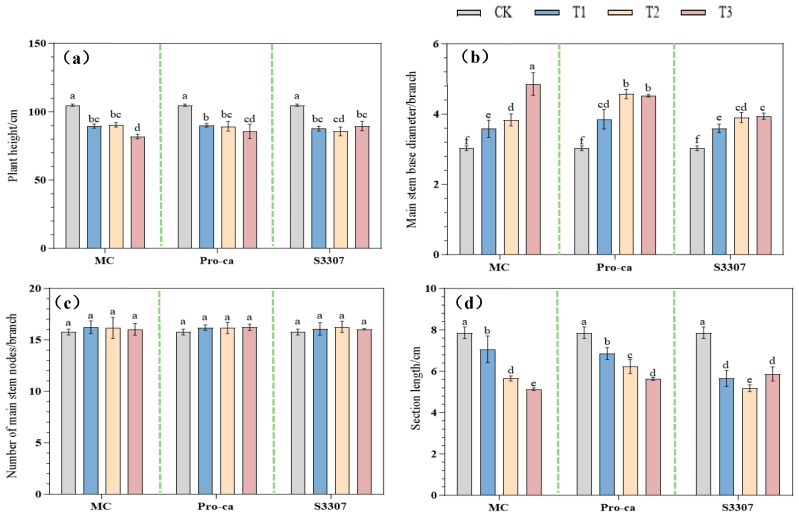
Effect of different plant growth regulators on (**a**) plant height, (**b**) main stem diameter, (**c**) number of nodes, and (**d**) internode length. Lowercase letters in the graphs indicate significant differences at *p* < 0.05. MC indicates 98% mepiquat chloride, with T1: 200 mg/L, T2: 250 mg/L, and T3: 300 mg/L; Pro-Ca indicates 5% prohexadione-calcium, with T1: 150 mg/L, T2: 250 mg/L, and T3: 350 mg/L; and S3307 denotes 5% uniconazole, with T1: 50 mg/L, T2: 100 mg/L, and T3: 150 mg/L.

As shown in Figure 1, the PGR treatments significantly affected plant height, internode length, and main stem diameter of purple-flowered alfalfa plants (*p* < 0.05); however, none of the treatments induced significant changes in the count of nodes along the primary stem (*p* > 0.05). Compared with CK, the MCT1, MCT2, and MCT3 significantly reduced plant height and spacing between the eighth and ninth nodes of the main stem (*p* < 0.05), with MCT3 resulting in the lowest values of 81.95 cm and 5.14 cm, respectively, corresponding to 22% reduction in plant height and 25.9% reduction in internode length. Under MCT3, the basal diameter of the main stem was the maximum at 4.86 mm, indicating an increase of 59.8% compared with CK. Compared with CK, the Pro-CaT1, Pro-CaT2, and Pro-CaT3 significantly reduced plant height and the spacing between the eighth and ninth nodes of the main stem (*p* < 0.05), with those under Pro-CaT3 being the lowest at 85.59 cm and 5.63 cm, respectively, corresponding to the reductions of 18.5% and 28.3%. Under Pro-CaT3, the basal diameter of the main stem was the largest at 4.52 mm, an increase of 50.1% compared with CK.

Similarly, S3307T1, S3307T2, and S3307T3 significantly reduced plant height and spacing between the eighth and ninth nodes of the main stem compared with CK (*p* < 0.05), with those under S3307T2 being the lowest at 85.64 cm and 5.17 mm, respectively, corresponding to the reductions of 18.4% and 25.4%. The basal diameter of the main stem was the largest under S3307T3, at 3.93 mm, indicating an increase of 29.2% compared with CK. No significant difference was observed in the count of nodes on the main stem among the groups (*p* > 0.05). Taken together, these results indicate that with the increase in PGR concentration, the diameter tended to increase in the MC group, while the internode length showed a decreasing trend in the MC and Pro-Ca groups.

#### 2.1.2. Change Trend of the SPAD Value Under Different Treatments

**Figure 2:**
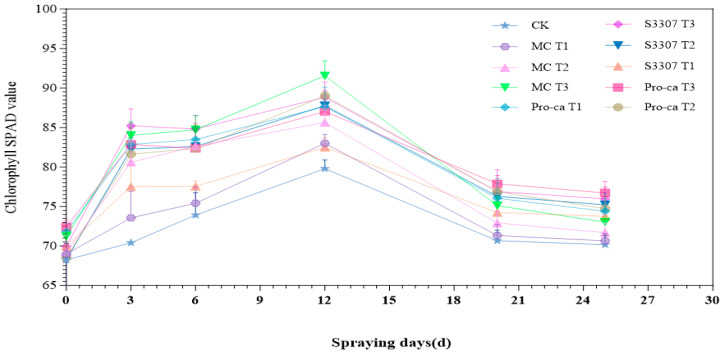
Temporal trends in chlorophyll SPAD values under treatments with different plant growth regulators. Error bars in the figure represent standard deviation (SD). Statistical analysis was conducted using one-way ANOVA combined with Duncan’s multiple comparison test with the significance level set at *p* < 0.05.

Within 0–25 days following the application of PGRs, the SPAD values of the treated alfalfa leaves exhibited a fluctuating trend—increasing, decreasing, and ultimately leveling off as the growth stage advanced (Figure 2). Specifically, the SPAD content peaked at 12 days after spraying (on 1 August). On the day of spraying (20 July), the SPAD values of the treated alfalfa leaves ranged from 68.22 to 71.21, showing no significant difference compared with CK (*p* > 0.05). On day 3 after spraying (on 23 July), the SPAD values of the treated alfalfa leaves ranged from 73.55 to 85.23, significantly higher than the CK (70.4); 6 days after spraying (on 26 July), the SPAD values of the treated alfalfa leaves ranged from 75.41 to 84.81, significantly higher than the CK (73.91); 12 days after spraying (on 1 August), marked by the peak flowering stage, the SPAD values of the treated alfalfa leaves ranged from 82.5 to 91.55, markedly higher than the CK (79.8); and 20 days after spraying (9 August), characterized by the maturity stage, the SPAD values of the treated alfalfa leaves ranged from 70.67 to 77.87, significantly lower than that during the peak flowering stage. Finally, 25 days after spraying (on 14 August), during the maturity stage, the SPAD values of the treated alfalfa leaves ranged from 73.01 to 76.72. Overall, SPAD values peaked at 12 days under all treatments. The maximum value was 91.55, as observed in the MCT3 group. These results indicate that the application of PGRs could effectively increase the chlorophyll content of alfalfa leaves and improve the photosynthetic efficiency, with 98% MCT3 (300 mg/L) having the most significant effect.

### 2.2. Effects of Different PGRs on the Seed Yield Components of Alfalfa

**Figure 3:**
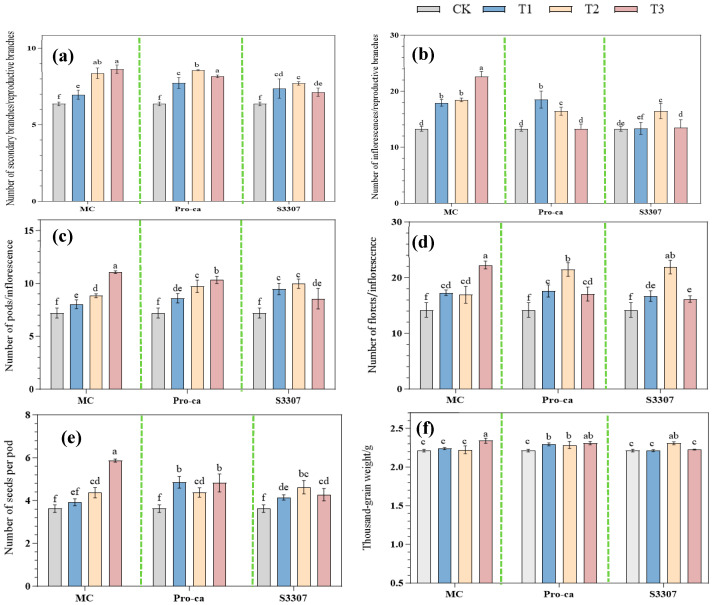
Effect of different plant growth regulators applied in different concentrations on (**a**) the number of secondary branches per reproductive branch, (**b**) the number of inflorescences per reproductive branch, (**c**) the number of pods per inflorescence, (**d**) the number of florets per inflorescence, (**e**) the number of seeds per pod, and (**f**) the thousand-seed weight of alfalfa. Lowercase letters in the graphs indicate significant differences at *p* < 0.05. MC indicates 98% mepiquat chloride; T1: 200 mg/L, T2: 250 mg/L, and T3: 300 mg/L; Pro-Ca indicates 5% prohexadione-calcium; T1: 150 mg/L, T2: 250 mg/L, and T3: 350 mg/L; S3307 denotes 5% uniconazole; and T1: 50 mg/L, T2: 100 mg/L, and T3: 150 mg/L.

Under all treatment groups, the number of secondary branches per reproductive branch, pods per inflorescence, seeds per pod, inflorescences per reproductive branch, and florets per inflorescence differed significantly from those in CK (*p* < 0.05). Although the thousand-seed weight (TSW) in most treatment groups showed no significant difference compared to the control group (*p* > 0.05), the TSW of plants treated with the highest concentration of mepiquat chloride (MC) was significantly lower than that of the control group (*p* < 0.05), indicating that this treatment had a noticeable impact on seed yield (Figure 3). The number of secondary branches per reproductive branch was significantly higher in the MC treatment groups (*p* < 0.05). The greatest number was observed in MCT3, with 8.63 branches, indicating a 35.47% increase compared with CK. MCT1 showed the lowest number of branches at 6.97, indicating a 9.41% increase compared with CK. The number of inflorescences per reproductive branch in all treatment groups was significantly higher than that in the CK (*p* < 0.05). No significant difference was observed between MCT1 and MCT2, with T3 exhibiting the highest number of inflorescences at 22.60, indicating a 69.92% increase compared with CK. The number of pods per inflorescence was also significantly higher in the treatment groups than in CK (*p* < 0.05), with the highest number observed under MCT3 at 11.07, indicating a 53.75% increase compared with CK. These results indicate a direct relation between the number of pods and MC concentration. In addition, the number of florets per inflorescence was significantly higher in all MC treatment groups (*p* < 0.05). No significant difference was observed between the MCT1 and MCT2 groups in terms of their response to the treatment; the highest number of florets was observed under MCT3 at 22.27, indicating a 56.83% increase compared with CK. The number of seeds per pod under MCT1 did not differ significantly from that in CK (*p* > 0.05); however, the MCT3 group showed the maximum number at 5.82, indicating 60.77% increase compared with CK. Similarly, MCT1 and MCT2 did not differ significantly from CK in terms of thousand-seed weight (*p* > 0.05), and the highest thousand-seed weight was observed under MCT3 at 2.34 g, representing a 5.88% increase compared with CK.

In the Pro-Ca groups, the number of secondary branches per reproductive branch was significantly higher than that in CK (*p* < 0.05), with the highest number observed under Pro-CaT2 at 8.57, indicating 34.53% increase relative to CK. The number of inflorescences per reproductive branch was significantly higher under both Pro-CaT1 and Pro-CaT2 than in CK (*p* < 0.05); however, it did not differ significantly between Pro-CaT3 and CK (*p* > 0.05). The number of inflorescences was the highest under Pro-CaT1, at 18.50, which signified a 39.09% increase compared with CK and indicated a decreasing trend with the increase in Pro-Ca concentration. The treatment groups also showed a markedly higher pod count per inflorescence compared with CK (*p* < 0.05), with Pro-CaT3 showing the highest number, with 10.33 pods, indicating 43.47% increase compared with CK. The number of florets per inflorescence did not differ significantly between the Pro-CaT1 and Pro-CaT3 groups (*p* > 0.05), and it was found to be maximum under Pro-CaT2, at 21.47, indicating an increase of 51.19% compared with CK. The quantity of seeds in each pod under all treatments was significantly higher than that in CK (*p* < 0.05), with the difference between Pro-CaT1 and Pro-CaT3 being nonsignificant (*p* > 0.05). The lowest number of seeds was observed under Pro-CaT2 at 4.38, which represented a 20.99% increase compared with CK. Thousand-seed weight showed a significant increase in all treatment groups compared with CK (*p* < 0.05). Specifically, Pro-CaT1, Pro-CaT2, and Pro-CaT3 showed 3.61%, 3.21%, and 4.50% increases relative to CK, respectively, with no significant intergroup differences (*p* > 0.05).

Paralleling the trends in the MC and Pro-Ca groups, the S3307 groups showed a significant increase in the number of secondary branches per reproductive branch compared with CK (*p* < 0.05). S3307T2 exhibited the highest number of secondary branches, at 7.70, representing a 21% increase compared with CK. The number of secondary branches was the lowest under S3307T3, with 7.13 branches, marking an 11.93% increase compared with CK. The number of inflorescences per reproductive branch in the S3307T2 group was significantly higher than that in CK (*p* < 0.05), reaching a maximum of 16.47, representing a 23.83% increase compared with CK. This number exhibited a downward trend with the increase in S3307 concentration. The S3307 treatment groups also showed a significantly higher number of pods per inflorescence compared with the control group (CK) (*p* < 0.05), with no significant difference observed between S3307T1 and S3307T2 (*p* > 0.05). The lowest number was observed under S3307T3, with 8.57 pods, representing a 19.03% increase compared with CK. The number of florets per inflorescence was significantly higher in the treatment groups than in the control group (CK) (*p* < 0.05), with no significant difference between S3307T1 and S3307T3 (*p* > 0.05). S3307T2 exhibited the highest number, with 21.9 florets, representing a 54.22% increase compared with CK. The number of seeds per pod was also significantly higher in all treatment groups compared with the control (*p* < 0.05), and the highest count was observed in S3307T2, with 4.62 seeds, marking an increase of 27.62% compared with CK. In terms of thousand-grain weight, S3307T1 and S3307T3 did not differ significantly from CK (*p* > 0.05), and the S3307T2 group exhibited the highest weight, at 2.31 g, an increase of 4.52% compared with CK. This indicates that the inhibitory effect of uniconazole on the plants was weakened at a high concentration (T3; 150 mg/L).

### 2.3. Effects of Different PGRs on the Seed Yield of Purple Alfalfa

**Figure 4:**
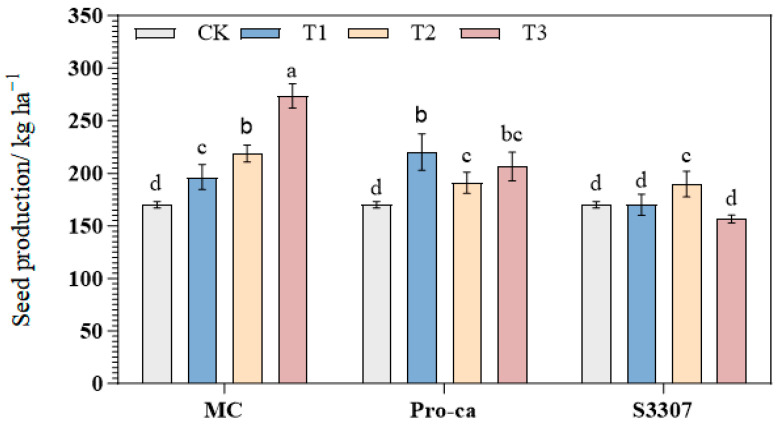
Effect of different plant growth regulators on alfalfa seed production. Different lowercase letters in the graphs indicate significant differences at *p* < 0.05. MC indicates 98% mepiquat chloride; T1: 200 mg/L, T2: 250 mg/L, and T3: 300 mg/L; Pro-Ca indicates 5% prohexadione-calcium; T1: 150 mg/L, T2: 250 mg/L, and T3: 350 mg/L; S3307 indicates 5% uniconazole; and T1: 50 mg/L, T2: 100 mg/L, and T3: 150 mg/L.

As shown in Figure 4, seed yield in the MC and Pro-Ca treatment groups differed significantly from that in CK (*p* < 0.05); however, the S3307 treatment groups, except for S3307T2, showed no significant difference from CK (*p* > 0.05). Seed yield in the 98% MC treatment groups increased as the treatment concentration was increased; the highest yield was obtained under MCT3 (300 mg/L), reaching 273.47 kg/ha, and showing an increase of 60.78% compared with CK. Among the Pro-Ca treatment groups, Pro-CaT1 exhibited the highest yield, reaching 220.10 kg/ha, and representing an increase of 29.40% over CK with no significant difference noted between Pro-CaT2 and Pro-CaT3 (*p* < 0.05). Seed yield in the S3307 treatment groups initially increased and then decreased with the increase in concentrations. It was found to be the highest under S3307T2 (100 mg/L), reaching 189.76 kg/ha, and indicating an 11.57% increase compared with CK.

### 2.4. Correlation Analysis of Three Plant Growth Regulators with Agronomic Traits and Yield Components

#### 2.4.1. Analysis of the Association Between 98% Mepiquat Chloride and Agronomic Traits as Well as Yield Components in Alfalfa

**Figure 5:**
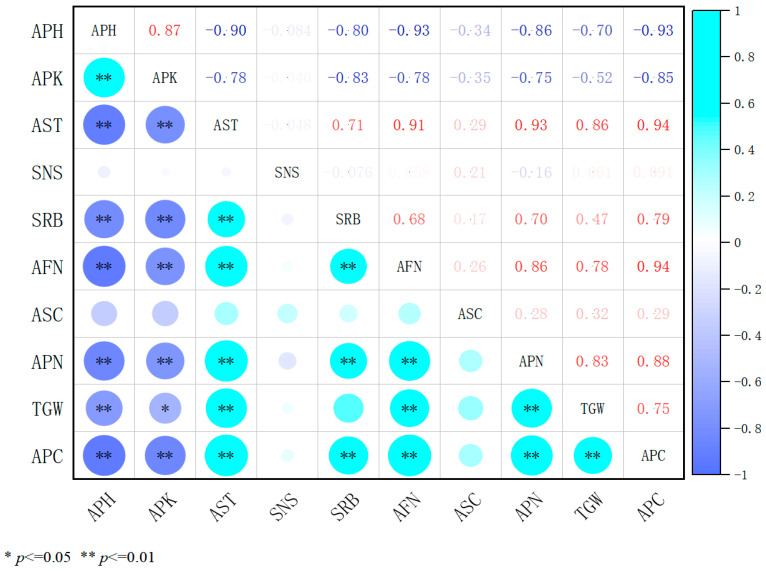
Coefficients of correlation between indicators under 98% mepiquat chloride treatments. APH, plant height; APK, internode length; AST, stem diameter; SNS, stem node number; SRB, secondary branching per reproductive branch; AFN, number of florets per inflorescence; ASC, number of seeds per pod; APN, number of pods per inflorescence; TGW, thousand-grain weight; and APC, seed yield. In the heatmap, darker colors indicate stronger correlations, while lighter colors indicate weaker ones.

Correlation analysis (Figure 5) following treatment with different concentrations of 98% mepiquat chloride (MC) demonstrated that plant height was significantly negatively correlated with seed yield, internode length, and the number of secondary branches on reproductive stems (*p* < 0.01), suggesting that increased plant height adversely affects seed yield and inhibits branching. In contrast, stem diameter exhibited a highly significant positive correlation with seed yield, the number of florets per inflorescence, and the number of pods per inflorescence (*p* < 0.01), highlighting the critical role of stem thickening and increased floret production in promoting seed yield. Thousand-grain weight showed a moderate positive correlation with seed yield, while internode length exerted suppressive effects on most traits. These findings indicate that, under 98% MC treatment, regulating plant height, increasing stem diameter, and enhancing floret numbers are pivotal strategies for optimizing seed yield. The results further confirm that taller plant structures are detrimental to achieving high seed production.

#### 2.4.2. Analysis of the Association Between 5% Prohexadione-Calcium and Agronomic Traits as Well as Yield Components in Alfalfa

**Figure 6:**
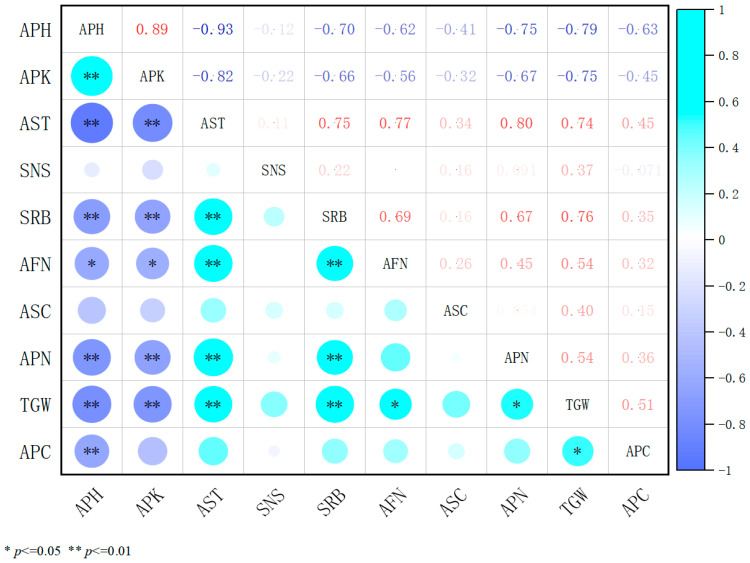
Coefficients of correlation among various indicators under 5% prohexadione-calcium treatments. APH, plant height; APK, internode length; AST, stem diameter; SNS, stem node number; SRB, secondary branching per reproductive branch; AFN, number of florets per inflorescence; ASC, number of seeds per pod; APN, number of pods per inflorescence; TGW, thousand-grain weight; and APC, seed yield. In the heatmap, darker colors indicate stronger correlations, while lighter colors indicate weaker ones.

Under 5% prohexadione-calcium treatment at varying concentrations, correlation analysis (Figure 6) revealed that stem diameter was positively correlated with the number of florets per inflorescence, pods per inflorescence, and thousand-grain weight, with significant links between floret and pod numbers. Secondary reproductive branch number also positively correlated with floret number, while plant height correlated with stem diameter (*p* < 0.05), suggesting that thicker stems enhance floret differentiation and pod development. Conversely, plant height was negatively correlated with internode length, branch number, and pod number (*p* < 0.05). Internode length was negatively correlated with stem diameter and pod number (*p* < 0.05), indicating that taller plants and elongated internodes suppress branching and pod formation, while shorter internodes enhance stem thickening. Stem node number and seeds per pod showed weak, nonsignificant correlations (*p* > 0.05), suggesting minimal impact on yield. Overall, 5% prohexadione-calcium improved alfalfa growth by regulating plant height, increasing stem diameter, and enhancing reproductive traits to optimize yield.

#### 2.4.3. Analysis of the Association Between 5% Uniconazole and Agronomic Traits as Well as Yield Components in Alfalfa

**Figure 7:**
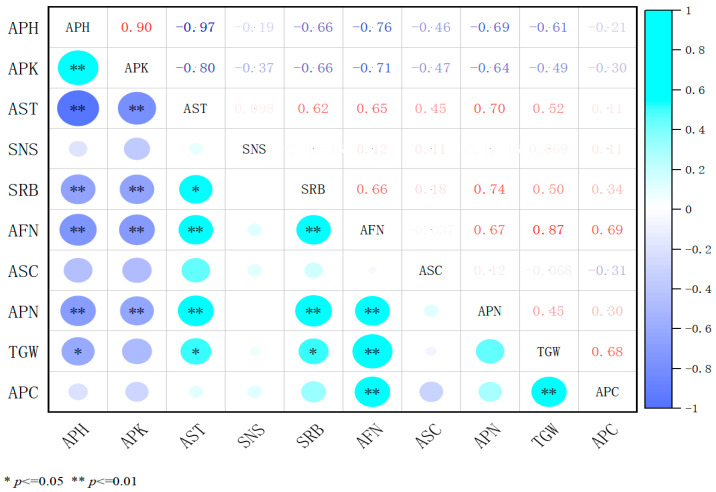
Coefficients of correlation among various indicators under 5% uniconazole treatments. APH, plant height; APK, internode length; AST, stem diameter; SNS, stem node number; SRB, secondary branching per reproductive branch; AFN, number of florets per inflorescence; ASC, number of seeds per pod; APN, number of pods per inflorescence; TGW, thousand-grain weight; and APC, seed yield. In the heatmap, darker colors indicate stronger correlations, while lighter colors indicate weaker ones.

After 5% uniconazole treatment, correlation heatmap analysis (Figure 7) revealed that alfalfa plant height was highly positively correlated with internode length (*p* < 0.01) and significantly negatively correlated with stem thickness and the number of pods per inflorescence (*p* < 0.05), indicating that taller plants with longer internodes inhibit stem thickening and pod formation. Stem thickness was highly positively correlated with the number of flowers per inflorescence, the number of pods per inflorescence, and thousand-seed weight (*p* < 0.01). Additionally, flower quantity was strongly associated with pod formation (*p* < 0.01). The number of secondary branches on reproductive stems was significantly positively correlated with flower quantity (*p* < 0.05), while plant height negatively affected branch development (*p* < 0.05). Internode length was significantly negatively correlated with stem thickness and pod formation (*p* < 0.05), suggesting that shorter internodes enhance stem thickening and pod development. The number of stem nodes and seeds per pod showed no significant correlations with other traits (*p* > 0.05), indicating their limited impact on yield. Seed yield was positively correlated with stem thickness, pod number, and thousand-seed weight (*p* < 0.01). In conclusion, 5% uniconazole improves seed yield by regulating plant height, shortening internodes, thickening stems, and promoting flower differentiation, providing a theoretical foundation for its application in high-yield alfalfa cultivation.

#### 2.4.4. An Integrated Correlation Analysis of the Effects of Three Plant Growth Regulators on the Traits and Yield Components of Alfalfa

**Figure 8:**
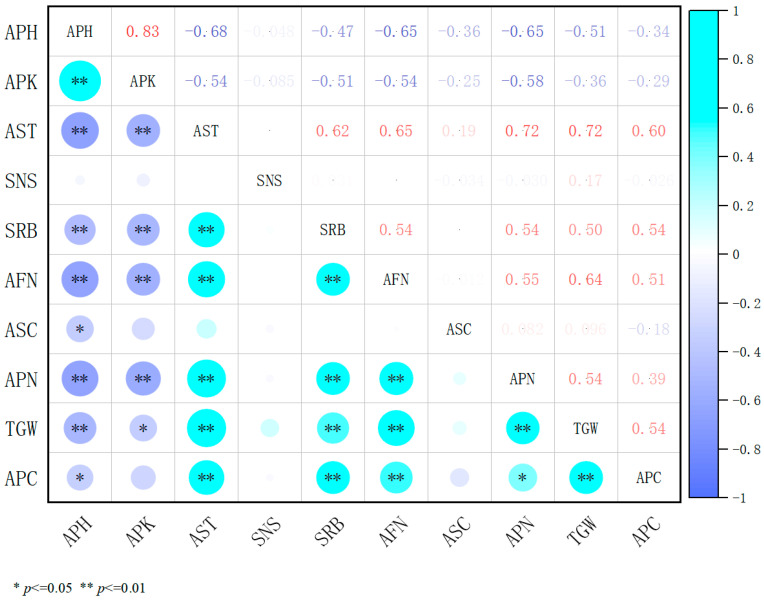
Correlation between treatment indicators. APH, plant height; APK, internode length; and AST, stem diameter: SNS, stem node number: SRB, secondary branching per reproductive branch; AFN, number of florets per inflorescence; ASC, number of seeds per pod; APN, number of pods per inflorescence; TGW, thousand-grain weight; and APC, seed yield. In the heatmap, darker colors indicate stronger correlations, while lighter colors indicate weaker ones.

Analysis of the comprehensive correlation heatmap (Figure 8) indicates that, under treatments with three plant growth regulators at different concentrations, plant height and internode length were highly positively correlated (*p* < 0.01), reflecting their shared role in promoting longitudinal growth. The number of pods per inflorescence showed a highly significant positive correlation with seed yield (*p* < 0.01), underscoring pod quantity as a key determinant of yield formation. Conversely, stem thickness and seed yield, as well as the number of stem nodes and seed yield, exhibited highly significant negative correlations (*p* < 0.01), suggesting that stem robustness and node number may influence yield through resource allocation trade-offs. Thousand-grain weight was positively correlated with seed yield (*p* < 0.05), though its contribution was less pronounced compared to pod number. In summary, alfalfa yield formation is governed by the interplay between plant architecture and reproductive traits. Appropriate regulatory measures can optimize resource allocation to disrupt unfavorable correlation trends, providing a theoretical foundation for the application of plant growth regulators in high-yield alfalfa cultivation.

### 2.5. Principal Component Analysis for a Comprehensive Evaluation of Seed Yield and Yield Components in Purple Alfalfa

Principal component analysis was performed on 10 indicators of purple alfalfa plants treated with different PGRs and under differed concentration gradients—plant height, internode distance, stem diameter, number of stem nodes, number of secondary branches per reproductive branch, number of florets per inflorescence, number of seeds per pod, number of pods per inflorescence, thousand-seed weight, and seed yield. Four principal components were extracted, namely PC1, PC2, PC3, and PC4, with variance contributions of 49.5%, 13.7%, 10.4%, and 7.2%, respectively. The cumulative contribution rate was 80.814%, which could explain the main factors related to agronomic traits and seed yield components. PC1, with an eigenvalue of 4.592, showed high loadings in its eigenvector for stem diameter, number of florets per inflorescence, and number of pods per inflorescence, mainly reflecting the seed yield of alfalfa. This underscored the relation of these factors with seed yield. PC2, with an eigenvalue of 1.368, exhibited high factor loadings for plant height and internode distance in alfalfa, underscoring their value as key agronomic traits. PC3, with an eigenvalue of 1.040, exhibited the highest absolute factor loading for the number of stem nodes, which can thus be considered a key agronomic trait. PCA4 showed the highest absolute factor loading for internode distance, highlighting its importance as a key agronomic trait. The eigenvectors were calculated on the basis of the eigenvalues of the PCs and the corresponding variance contribution rates (Table 1). These eigenvectors were used as coefficients to construct the functional expressions of the PCs:

F_1_ = −0.375X_1_ − 0.338X_2_ + 0.401X_3_ + 0.025X_4_ + 0.335X_5_ + 0.364X_6_ + 0.077X_7_ + 0.359X_8_ + 0.346X_9_ + 0.291X_10_

F_2_ = 0.338X_1_ + 0.315X_2_ + 0.029X_3_ + 0.009X_4_ + 0.175X_5_ + 0.123X_6_ − 0.714X_7_ − 0.38X_8_ + 0.159X_9_ + 0.451X_10_

F_3_ = −0.012X_1_ − 0.049X_2_ − 0.054X_3_ + 0.970X_4_ − 0.033X_5_ − 0.002X_6_ − 0.042X_7_ − 0.101X_8_ + 0.176X_9_ − 0.090X_10_

F_4_ = 0.202X_1_ + 0.510X_2_ + 0.295X_3_ − 0.028X_4_ − 0.205X_5_ − 0.071X_6_ + 0.539X_7_ − 0.174X_8_ + 0.496X_9_ + 0.207X_10_.

**Figure 9:**
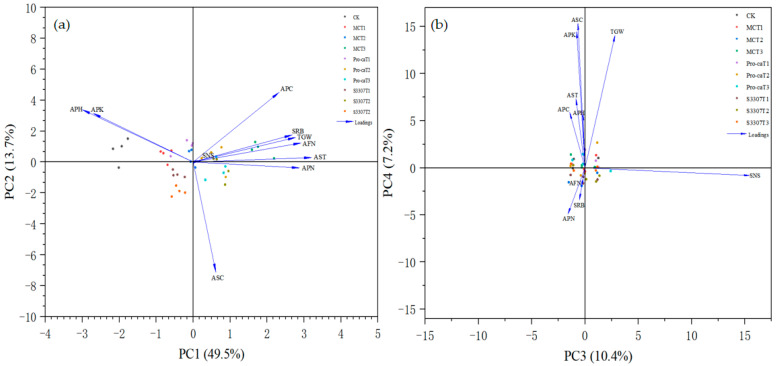
Principal component analysis plots: (**a**) PC1 vs. PC2 and (**b**) PC3 vs. PC4, respectively illustrating the relationships between samples and loading vectors, along with their total contribution rates. APH, plant height; APK, internode length; AST, stem diameter: SNS, stem node number; SRB, secondary branching per reproductive branch; AFN, number of florets per inflorescence; ASC, number of seeds per pod; APN, number of pods per inflorescence; TGW, thousand-grain weight; and APC, seed yield.

Standardizing the raw SPSS data, common factors were extracted. These factors were substituted into the equation Y = 0.452F1 + 0.1368F2 + 0.1040F3 + 0.0719F4. Finally, a comprehensive evaluation model was constructed (Figure 9).

The comprehensive scores for different PGR treatments are shown in Table 2. Accordingly, the treatments were ranked as follows: MCT3 > Pro-CaT3 > S3307T2 > Pro-CaT2 > Pro-CaT1 > MCT2 > S3307T3 > S3307T1 > MCT1 > CK. According to the model and the functional expression, the comprehensive score in terms of the factors related to agronomic traits and seed yield components of purple alfalfa was the highest under MCT3.

## 3. Discussion

This study identified the most suitable PGRs and optimum concentration ranges based on changes in various indicators of plant height and seed yield components in purple alfalfa. As reported, alfalfa exhibits a vigorous vegetative growth pattern, which suppresses its reproductive growth, leading to a reduction in seed yield [34]. Specifically, alfalfa crops exhibit an indeterminate growth pattern, with the vegetative growth sustaining even during the reproductive phase, intensifying the competition for nutrients. In addition, the plant height of alfalfa is an important indicator of yield and is closely related to production [35]. PGRs are widely recognized for their influence on plant development and growth. Plant height variation is usually caused by changes in the number or length of internode cells [36]. This is consistent with the findings of the current study. Plant height is a key indicator of the growth status of forage grasses [37,38]. The introduction of semi-dwarf and dwarf strains of rice and wheat, respectively, during the “Green Revolution” underscores the significance of plant height in regulating crop production. A dwarf phenotype significantly enhances lodging resistance in plants and is thus fundamental to high crop yields [39,40]. However, crop yield does not necessarily increase with plant height; excessive vegetative growth can seriously hinder reproductive growth, reduce quality, and pose harvesting challenges. In cotton, a significant negative correlation has been reported between plant height and seed yield [41]. In the present study, the use of PGRs significantly increased stem thickness and enhanced the yield of alfalfa seeds. This may be attributed to the PGR treatment-induced development of a complete vascular bundle system in the stems, which provides increased space for nutrient transport and the accommodation of more phloem and xylem structures, thus enhancing the transport flux and the transport of organic matter, water, and minerals, which are crucial for meeting the growth demands of alfalfa plants. In maize, MC (250 mg/L) treatment was reported to reduce plant height, increase stem diameter and lignin content, and enhance lodging resistance, effectively improving maize yield [42,43]. This suggests that stem size is closely related to yield. The application of MC during the crop growth stage improves the source–sink relationship, triggering the synthesis of carbohydrates, reducing the allocation of photosynthates to the main stem, branches, and apical growth points, and increasing resource allocation to the reproductive organs [44]. The use of Pro-Ca has been shown to suppress stem elongation and thickening and cause an increase in floral count and fruit production [45]. Additionally, foliar application of Pro-Ca was reported to enhance the photosynthetic capacity [46,47]. Its application significantly reduced the height of hyacinth bean plants and increased seed weight, which highlighted its role as a promising agricultural production strategy. Uniconazole (S3307) reduces plant height; however, at high doses, it inhibits stem elongation, affecting grain yield, panicle length, and corn cob weight, with stem diameter decreasing as the concentration of uniconazole increases [48]. In addition, the application of MC and Pro-Ca in optimal concentrations has been reported to cause reductions in internode length and nutrient growth and an increase in stem thickness [49,50]. This aligns with the findings of the current research. Uniconazole application was also reported to prevent vegetative growth in soybeans during the blooming phase period and extend the life of photosynthetically active leaves during the pod-setting stage, eventually leading to an increase in yield [51]. MC, Pro-Ca, and uniconazole are commonly employed PGRs that effectively promote plant growth and development by inhibiting the synthesis of gibberellins within plants [52], slowing down the cell growth rate, shortening internode length and thus reducing plant height [53], and inhibiting vegetative growth to increase stem diameter and seed yield.

To our knowledge, no study has explored the impacts of PGR treatments on the yield of alfalfa seeds, a valuable forage crop. In contrast, traditional methods of alfalfa seed production failed to fully exploit the plant’s growth potential, particularly after multiple harvests, resulting in extended production cycles. This study underscores the importance of PGR application during post-harvest alfalfa seed production not only in mitigating economic losses associated with decreased forage yield and quality due to increased harvesting frequency, but also in addressing regional seed deficits. At the same time, this study provides a reference for the types of growth hormones and their optimized concentrations suitable for seed production in arid and semi-arid regions. This study demonstrated that the use of MC, Pro-Ca, and S3307 inhibited excessive growth of purple alfalfa plants by decreasing the internode length, reducing plant height, and enhancing factors related to stem diameter and seed yield, such as secondary branching, small inflorescences, thousand-grain weight, the number of pods, and seed yield. These findings suggest that by preventing excessive growth of alfalfa, PGRs optimize plant height and induce a shift from the vegetative to the reproductive phase, consequently enhancing seed yield components and maximizing seed yield. Further research is required to determine the optimal combination of PGRs and confirm whether combining chemical treatment with physical methods to eliminate apical dominance (e.g., topping) can more effectively regulate plant morphology and yield potential in alfalfa. In-depth studies are warranted to elucidate the pathways and effects of PGRs on the signal transduction process in alfalfa, which can facilitate the development of efficient and high-yielding alfalfa seed production technology.

## 4. Materials and Methods

### 4.1. Overview of the Experimental Site

**Figure 10:**
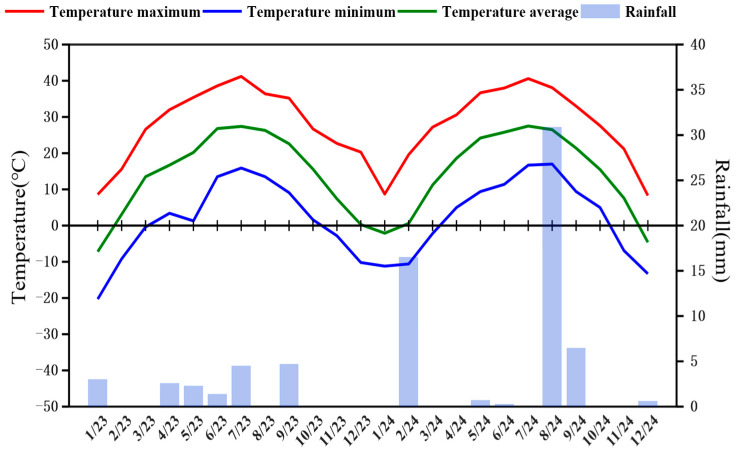
Maximum and minimum temperatures, average temperatures, and monthly rainfall at the test site during 2023–2024.

The experimental site is located at the Alfalfa Science and Technology Yard in Luopu County, Hotan Region, Xinjiang (37°14′02″ N, 80°12′02″ E, altitude 1344 m). The region has sandy saline–alkaline soil and is situated at the junction of the alluvial fan at the foot of the mountains and the northern desert region. It exhibits abundant light and heat resources, with an annual sunshine duration of 2653.7 h, an average temperature ranging from 7.8 to 12 °C, daily temperature difference averaging 13.9 °C (Figure 10), a frost-free period of 217 days, annual precipitation averaging 35.2 mm, and an annual evaporation of 2226.2 mm.

### 4.2. Experimental Materials

The “Xinmu No. 5” cultivar of alfalfa (*M. sativa* L.) provided by the College of Grassland Science, Xinjiang Agricultural University, was used as the experimental material. Three test agents were used in the experiment, and their specification, active ingredient, and manufacturer information are presented in Table 3.

### 4.3. Experimental Design

This study employed a randomized block design, using the surface sprinkler irrigation method. The area of each plot was 12 m^2^ (3 m × 4 m), featuring a row spacing of 60 cm and a plant spacing of 20 cm, and a 1 m interval between adjacent plots. Purple-flowered alfalfa plants were treated with water-dispersible granules (5%) of uniconazole (S3307), soluble powder formulation of 98% MC, and wettable powder formulation of 5% Pro-Ca in July 2024 when the plants were in the branching and initial flowering stages. Each PGR was tested at three concentrations: T1, T2, and T3. Specifically, MC was applied in 200 mg/L (MCT1), 250 mg/L (MCT2), and 300 mg/L (MCT3) concentrations; Pro-Ca was applied in 150 mg/L (Pro-CaT1), 250 mg/L (Pro-CaT2), and 350 mg/L (Pro-CaT3) concentrations; and S3307 was applied in 50 mg/L (S3307T1), 100 mg/L (S3307T2), and 150 mg/L (S3307T3) concentrations. Additionally, an equal amount of clear water (CK) was used as the control, resulting in 10 treatments, each with 3 replicates, totaling 30 plots. The volume of the spray solution was set at 150 mL/m^2^ for all treatments. Spraying was conducted twice during the branching and initial flowering stages between 8:00 and 11:00 AM on clear, windless mornings. A handheld sprayer was used for foliar application. During spraying, each plot was shielded with a board to prevent drift, and conventional field management was maintained throughout the experiment.

### 4.4. Measurement Indices and Methods

#### 4.4.1. Alfalfa Growth Traits

At maturity, 10 alfalfa plants were randomly selected from each plot for measurements, which included height, main stem nodes, internode length, and basal diameter of the main stem. The results were averaged.

Determination of soil plant analysis development (SPAD) values: SPAD values were measured using a handheld portable chlorophyll meter (Beijing Zhongke Weihe Technology Development Co., Ltd., Beijing, China). Measurements were taken on the day of spraying (20 July), 3 days after spraying (23 July), 6 days after spraying (26 July), 12 days after spraying (1 August), 20 days after spraying (8 August), and 25 days after spraying (13 August) during sunny conditions between 9:30 AM and 12:00 PM. The SPAD values of the middle top leaves of the second-highest level of the primary alfalfa plants were determined. Ten leaves with consistent growth condition and light exposure direction were selected randomly from the treatment plots for measurement, and the results were averaged.

#### 4.4.2. Determination of Seed Yield Components

Number of secondary branches per reproductive branch: The number of secondary branches was counted in each of the 30 reproductive branches selected randomly from each treatment group during the full bloom stage.

Number of inflorescences per reproductive branch: The number of inflorescences was counted in each of the 30 reproductive branches selected from each treatment group during the full bloom stage.

Number of florets per inflorescence: This parameter was determined by randomly selecting 30 inflorescences from different parts of the chosen reproductive branches during the full bloom stage.

Number of pods per inflorescence: The number of pods was counted in each of the 30 plants selected randomly at maturity from each treatment group.

Number of seeds per pod: The number of seeds was counted in pods from different parts of each of the 30 alfalfa plants selected randomly from each treatment group after 80% of the pods turned dark brown.

Thousand-seed weight: After processing the harvest from each treatment group uniformly, the cleaned seeds from each group were dried. Then, 1000 clean seeds were randomly separated, and this process was repeated 4 times. The weight of the seeds was measured, and the values for each group were averaged.

Seed yield: When 80% of the pods in each treatment group turned dark brown, the plants were cut at ground level, bagged, and sun-dried. Subsequently, the seeds were threshed and cleaned manually and then weighed using a 1/1000 precision scale. Then, the yield per hectare was calculated.

### 4.5. Data Statistics and Analysis

The dataset was statistically analyzed using Excel 2019. One-way analysis of variance and Duncan’s multiple range test were performed in SPSS 26.0 to compare the results of different plant growth regulator (PGR) treatment groups with those of the control group (CK). Principal component analysis and correlation analysis were conducted using Origin2024, and bar charts and line graphs were generated using GraphPad Prism 10.1.0. Additionally, MATLAB was used to create climate graphs.

## 5. Conclusions

Foliar application of 300 mg/L mepiquat chloride (MCT3) significantly reduced plant height and internode length by 22% and 25.9%, respectively, while promoting basal stem diameter growth and optimizing reproductive conditions. This treatment suppressed excessive vegetative growth, enhanced nutrient conversion and utilization, and improved reproductive development. Twelve days after application, the SPAD value reached 91.55%, the highest among all treatments. MCT3 also significantly increased secondary branches, florets, pods per inflorescence, seeds per pod, thousand-seed weight, and seed yield, resulting in a 60.78% increase in seed yield. Principal component analysis confirmed MCT3 as the most effective treatment, highlighting its potential for improving alfalfa seed yield and quality.

The application of MCT3 significantly improved the yield and quality of alfalfa seeds by regulating plant morphology, growth and development, nutrient allocation, and reproductive status. These findings provide practical value and new strategies for enhancing alfalfa seed production. Future research should further explore the molecular mechanisms of mepiquat chloride, including its effects on nutrient transport, photosynthetic efficiency, and the expression of key genes related to seed yield. Additionally, it is necessary to evaluate its regulatory effects under different environmental conditions (such as drought or salt stress) to provide comprehensive guidance for alfalfa management in arid and semi-arid regions.

## Figures and Tables

**Table 1:** Eigenvectors of each index in each principal component.

Name	PCA1	PCA2	PCA3	PCA4
Plant height	−0.37482	0.33828	−0.01204	0.20175
Internode length	−0.33816	0.31489	−0.04906	0.51011
Stem diameter	0.40061	0.02864	−0.05374	0.25876
Stem nodes	0.02495	0.00859	0.97031	−0.0288
Secondary branching	0.33465	0.1755	−0.0335	−0.1206
Simple inflorescence	0.36452	0.12324	−0.02129	−0.07091
Number of seeds per pod	0.07694	−0.71337	−0.04231	0.54017
Number of pods per inflorescence	0.35885	−0.03799	−0.10129	−0.17392
Thousand-grain weight	0.34607	0.15899	0.17672	0.4967
Seed production	0.29106	0.45157	−0.09057	0.20733
Eigenvalue	4.952	1.368	1.040	0.719
Individual contributions	49.523	13.686	10.405	7.199
Cumulative contribution	49.523	63.209	73.61484	80.814

**Table 2:** Comprehensive score for different treatments.

Treatment	F1	F2	F3	F4	Y
CK	−4.385	0.385	−0.25	0.495	−2.110
MCT1	−1.652	0.175	0.235	0.078	−0.763
MCT2	−0.975	0.133	−0.227	−0.758	−0.108
MCT3	4.015	0.910	−0.260	0.380	2.110
Pro-CaT1	−0.463	0.835	0.280	0.673	−0.038
Pro-CaT2	1.315	0.333	−0.163	0.310	0.703
Pro-CaT3	1.473	−0.14	0.218	0.493	0.770
S3307T1	−0.907	−0.828	−0.135	−0.980	−0.678
S3307T2	1.670	−0.545	0.355	−0.685	0.740
S3307T3	−0.907	−1.2575	0.050	−0.0025	−0.625

**Table 3:** Details of plant growth regulators.

Name	Specification	Active Ingredient	Factory
Mepiquat	5 g.bag	98% (C_7_H_16_NCl)	Sichuan Guoguang Agrochemical Co. (Chengdu, China)
Prohexadione-calcium	10 g.bag	5% (2(C_10_H11O_5_)•Ca	Anyang Quanfeng Biotechnology Co. (Anyang, China)
Uniconazole	10 g.bag	5% (C_15_H_18_ClN_3_O)	Sichuan Guoguang Agrochemical Co. (Chengdu, China)

## Data Availability

The data that support the findings of this study are available from the corresponding author, S.Z., upon reasonable request.
